# Knowledge and practices of anti-malarial treatment for children under five years among village health teams in Kasese District, Uganda: A cross-sectional study

**DOI:** 10.1016/j.ijregi.2025.100749

**Published:** 2025-09-08

**Authors:** Nakitto Irene Kisakye, Helena C. Maltezou, Robert Mugarura, Arthur Nek Jonathan, Charles Etyang

**Affiliations:** 1 Department of Community Health, Kabale University, Kabale District, Uganda; 2 Directorate for Research, Studies and Documentation, National Public Health Organization, Athens, Greece; 3 Department of Surgery, Kabale University, Kabale District, Uganda; 4 Department of Nursing, Mbarara University of Science and Technology, Mbarara, Uganda

**Keywords:** Malaria, Village health teams, Young children, Antimalarial drugs, Uganda

## Abstract

**Objectives:**

Malaria remains a leading cause of morbidity and mortality among children under 5 in Uganda, particularly in rural and hard-to-reach areas. Village health teams (VHTs) play a pivotal role in community-based malaria diagnosis and treatment, yet their knowledge and practices remain understudied. To assess the knowledge and practice of antimalarial drug use among VHTs for children under 5 years in Kasese District, Uganda.

**Methods:**

A descriptive cross-sectional study was conducted among 102 VHTs using a structured, interviewer-administered questionnaire. Descriptive statistics were used to summarize VHT characteristics, knowledge, and practice scores. Chi-square tests were performed to assess associations between socio-demographic characteristics and key dispensing practices. Data were analyzed using SPSS, version 26.

**Results:**

The majority of VHTs demonstrated basic knowledge in antimalarial treatment, with 70.6% routinely checking expiration dates and 76.5% instructing caregivers on when to administer medications. However, fewer VHTs informed caregivers about side effects (21.6%), drug interactions (29.4%), or the effects of herbal medicines (19.6%). Significant associations were found between educational level and multiple dispensing practices (*p* <0.05), including giving drugs based on rapid diagnostic test results and appropriately addressing caregiver requests.

**Conclusions:**

While VHTs in Kasese District generally adhere to key elements of antimalarial drug administration, gaps persist in their pharmacological knowledge and patient education practices. Strengthening ongoing training and supervision may improve rational antimalarial drug use and treatment outcomes in children under 5 years old.

## Highlights

• Village health teams had good basic knowledge of malaria treatment.
• Few village health teams informed caregivers about drug side effects.
• Education level influenced prescribing practices.
• Gaps existed in counseling and drug interaction.
• Training needed to improve rational antimalarial use.

## Introduction

Malaria remains one of the most significant public health challenges in Uganda, particularly among children under 5 years of age. According to the World malaria report, malaria cases in Uganda increased from 1,502,362 in 2021 to 3,631,939 in 2022, with an increase of 52.5% mortality, including mortality among children below 5 years [1]. Additionally, Uganda is ranked the third country globally in terms of malaria burden, accounting for approximately 5.1% of global burden in 2021 [2]. Malaria is responsible for a considerable proportion of outpatient visits, hospital admissions, and deaths among children under 5 years. Given its widespread burden, timely diagnosis and prompt treatment with effective antimalarial agents are critical for reducing morbidity and mortality [2].

To improve access to timely antimalarial treatment in remote areas, Uganda adopted the village health team (VHT) strategy in 2001. VHTs serve as the first point of contact for basic health services in rural and underserved areas. As part of the World Health Organization’s (WHO) integrated community case management (iCCM) strategy, VHT responsibilities include identifying symptoms, using rapid diagnostic tests (RDTs), dispensing appropriate medications, and referring severe cases to health facilities [3]. The Government of Uganda, in collaboration with implementing partners, has provided VHTs with job aids, training manuals, and regular supply of medicines. However, studies have shown that the quality of care provided by VHTs varies, and concerns have been raised about their adherence to treatment guidelines and rational drug use [4,5]. Previous evaluations have documented challenges such as inconsistent supervision, inadequate refresher training, and limited understanding of drug interactions, adverse effects, and appropriate counseling [6].

Despite the strategic importance of VHTs in malaria control, there is limited recent data on their knowledge and practices regarding antimalarial treatment for children under 5 years. This gap in knowledge may hinder the effectiveness of community-based interventions, especially in high-transmission settings like Kasese District, which borders the Democratic Republic of Congo and frequently reports malaria outbreaks [7,8].

This study aimed to assess the knowledge and practice of VHTs regarding antimalarial drug use for children under 5 years of age in Kasese District, Uganda. Understanding these practices is crucial for identifying gaps, informing training needs, and enhancing the impact of community-level malaria control programs.

## Methods

A descriptive cross-sectional study was conducted in Kasese District, Western Uganda, from November to December 2024. Eligibility required at least 1 year of active service and involvement in malaria case management.

A structured questionnaire covered socio-demographic data, knowledge of antimalarial drug use, dispensing practices, and counseling behaviors. Associations between demographic variables and key practices were evaluated using chi-square or Fisher’s exact tests. Data were analyzed using SPSS version 26.

### Study setting

The study was conducted in Kasese District, Western Uganda, known for high malaria transmission. Data were collected from five sub-counties with active VHT networks: Maliba, Nyakiyumbu, Kisinga, Ihandiro, and Kitswamba.

### Study design and sampling

Cluster sampling was used to recruit a representative sample of VHTs in the district. A cluster of five sub-counties was randomly selected from the sampling frame of 39 sub-counties that made up the district [9]. A list of all VHTs was obtained in each of the selected sub-counties from the district health office. Probability proportionate sampling was used to get sample size for each of the selected sub-counties. Recruitment phone calls were made to each VHT, until a sample of 102 VHTs was successfully recruited.

### Sample size justification

The sample size was calculated using the following formula for a known population, the Cochran [10] formula ensured that the study on VHTs in Kasese District had a robust, representative sample that yielded reliable and generalizable data regarding their knowledge and practice in the rational use of antimalarial drugs for children under 5 years. A 95% confidence level and 5% margin of error was considered, resulting in a required sample of 102.

### Data collection tools

A pretested, structured, interviewer-administered questionnaire was used to collect data. The tool captured socio-demographics, knowledge of antimalarial use, and dispensing practices.

### Ethical considerations

Approved by Kabale University Research Ethics Committee (KABREC-2024-184). Administrative clearance obtained from the Kasese District Health Office. Written informed consent was obtained from all participants. Confidentiality was assured, and no personal identifiers were collected.

### Limitations of the study

The cross-sectional nature of the study limits causal inference. Additionally, self-reported practices may be subject to social desirability bias.

## Results

Of the 137 active VHTs in Kasese District, 102 VHTs met the inclusion criteria and participated, representing 74.4% of the total VHT population. All 102 eligible VHT members participated in the study (response rate: 100%). The educational qualifications of participants across all the sub-counties showed that the majority had secondary as the highest educational level attained (47.1%), followed by primary (25.5%), and tertiary (19.6%). The least number of participants were found to have university as the highest educational level attained (7.8%). Table 1 shows the respondents’ level of education.

**Table 1:** Respondents level of education (N = 102).

Highest educational level	n = 102 (%)
Primary	**26 (25.5)**
Secondary	**48 (47.1)**
University	**8 (7.8)**
Tertiary	**20 (19.6)**

Source: Researcher, 2025.

Table 2 shows their knowledge and practices about antimalarial treatment for children under 5 years old.

**Table 2:** Knowledge and practices about the rational use of antimalarial drugs for children under 5 years among participating VHTs, Kasese, Uganda, 2024 (N = 102).

Variable	Number of VHTs (n = 102) (%)
Check the expiration dates	72 (70.6)
Instruct the parents/guardians when to take the drugs	78 (76.5)
Instruct parents/guardians how to take the drugs (oral/rectal)	84 (82.4)
Instruct parents/guardians on drug dosage	74 (72.5)
Know drug indication	58 (56.9)
Store the drugs as recommended by their supervisors	62 (60.8)
Read package inserts	30 (29.4)
Instruct about food-drug interactions	24(23.5)
Instruct on treatment duration	26 (25.5)
Inform the parents about possible drug side effects	22 (21.6)
Know drug-drug interactions	30 (29.4)
Understand that herbal products and possible interactions with antimalarials	20 (19.6)

VHT, village health team.

Source: Researcher, 2025.

Table 3 shows the associations between the VHTs characteristics and practices of dispensing antimalarial drugs. Education level was significantly associated with prescribing after RDT (*p* < 0.001), response to caregiver demands (*p* = 0.03), and giving drugs to older children (*p* = 0.01). Female VHTs were more likely to follow RDT confirmation (*p* = 0.01).

**Table 3:** Bivariate analysis between characteristics and village health teams’ practices of dispensing antimalarial drugs, Kasese, Uganda.

Variable	Return unused drugs	Rapid diagnostic test-based prescribing	Dispense on request	Drugs for children above 5 years
Sex	0.95	0.01^a^	0.51	0.35
Level of education	0.62	0.00^a^	0.03^a^	0.01^a^
Sub-county	0.00^a^	0.82	0.28	0.01^a^
Years of service	0.04^a^	0.53	0.72	0.10

Source: Researcher, 2025.

a Indicates statistical significance.

## Discussion

This study assessed the knowledge and practices of VHTs in the management of malaria among children under 5 years in Kasese District, Uganda. Overall, the findings revealed that while a majority of VHTs had good basic knowledge of antimalarial drug use, significant gaps exist in their understanding of more advanced pharmacological and safety practices. For instance, although the majority of VHTs reported routinely checking drug expiration dates and explaining when and how to administer medications, only 19.6% were aware of potential drug interactions or the impact of herbal remedies. This demonstrates a partial understanding of rational drug use, which can compromise treatment effectiveness and safety. Notably, fewer than one-third of the respondents reported informing caregivers about possible side effects or the duration of treatment, which may affect adherence and outcomes.

These findings are consistent with similar studies in Uganda and other sub-Saharan African countries. For example, a study in Western Uganda found that patients at Sheema District Secondary Care Hospital had limited knowledge about their care, and the quality of prescriptions and services fell short of WHO standards [11]. This highlights a pressing need for continued educational programs targeting VHTs in Kasese, Uganda, to enhance proper drug prescribing, dispensing, and overall patient understanding. A 2022 study in Ghana found that only 23% of community health workers (CHWs) provided complete information on side effects, similar to the 21.6% figure in Kasese [12]. Another study in Nigeria reported that 38.7% of CHWs had inadequate knowledge of contraindications in malaria therapy, reinforcing the need for better pharmacological training [13].

The implications of our findings are critical. Inadequate understanding of adverse effects and drug interactions may contribute to suboptimal outcomes or adverse drug events, particularly in children under 5 years who are vulnerable to dosing errors and drug toxicity [14]. There is a pressing need to strengthen VHT training curricula to include modules on comprehensive pharmacological education, with emphasis on medication safety, patient counseling, and recognition of adverse events. To address the observed gaps, we recommend that the Ministry of Health and district health offices consider integrating regular refresher training sessions for VHTs.

A strength of this study lies in its focus on a hard-to-reach rural population and its relatively large sample size, which enhances the generalizability of findings within Kasese and similar districts. However, certain limitations must be acknowledged. The use of self-reported data may introduce social desirability bias, leading to overreporting of ideal practices. In addition, the cross-sectional design does not allow for causal inference between socio-demographic factors and observed knowledge or practices.

In conclusion, while VHTs in Kasese District, Uganda play a vital role in expanding access to antimalarial treatment, particularly for children under 5 years of age, their knowledge and practices reveal areas for improvement. Addressing the identified gaps through targeted training, regular supervision, and supportive policy frameworks is essential to achieving better treatment outcomes and reducing malaria-related morbidity and mortality among young children in Uganda.

## Recommendations

We recommend that the Ministry of Health, through the National Malaria Control Division, conduct routine refresher trainings for VHTs focused on pharmacovigilance, counseling, and rational drug use.

Kasese District Health Office should enhance community supervision systems to ensure VHTs adhere to national malaria treatment guidelines.

## Declaration of competing interest

The authors no have no competing interests to declare.
